# Langmuir Monolayer Studies of Phosphatidylcholine Membranes with Naproxen on the Polysaccharide Subphase

**DOI:** 10.3390/molecules30071509

**Published:** 2025-03-28

**Authors:** Małgorzata Jurak, Katarzyna Pastuszak, Agnieszka Ewa Wiącek

**Affiliations:** Department of Interfacial Phenomena, Institute of Chemical Sciences, Faculty of Chemistry, Maria Curie-Skłodowska University, Maria Curie-Skłodowska Sq. 3, 20-031 Lublin, Poland; katarzyna.pastuszak2@mail.umcs.pl (K.P.); agnieszka.wiacek@mail.umcs.pl (A.E.W.)

**Keywords:** phospholipid, chitosan, hyaluronic acid, nonsteroidal anti-inflammatory drug, Langmuir technique, Brewster angle microscopy, monolayer stability

## Abstract

Natural polysaccharides are biocompatible and biodegradable; therefore, they can be widely used in drug delivery, tissue engineering and wound healing. In this context, the interactions between polysaccharides, drugs and biological membranes are of great interest. In this paper, a 1,2-dipalmitoyl-*sn*-glycero-3-phosphatidylcholine (DPPC) monolayer was used as a model membrane to study the interactions with polysaccharides: chitosan (Ch) and/or hyaluronic acid (HA) and a nonsteroidal anti-inflammatory drug (NSAID) naproxen (NAP). The changes in the physicochemical properties of the model membrane were characterized by means of the Langmuir monolayer technique combined with Brewster angle microscopy (BAM). Compression/adsorption isotherms and morphology images were obtained at 20 °C. They allowed us to determine the effect of the subphase type (Ch, HA, Ch–HA) on the behavior of DPPC monolayers in the absence and presence of NAP, their elasticity, morphology and stability as a function of time. A potential mode of interactions between the phospholipid, polysaccharides and drug responsible for the change in membrane properties was proposed. These interactions regulate the efficiency of drug delivery systems, being of importance for living organisms in pain relief and wound healing.

## 1. Introduction

Chronic wounds are one of the most important health problems and are undoubtedly a significant cause of death worldwide. The use of appropriate dressings is essential to support wound healing. Among them, hydrogel-based products are very popular, which, owing to their properties, can significantly shorten the healing time and reduce the risk of recurrent infections. Various additives to the composition of hydrogels that can accelerate wound regeneration and minimize the feeling of pain are sought [1,2,3,4,5,6]. Many efforts are also being made to generate intelligent solutions that enable improvement of the properties of dressings, e.g., inhibiting bacterial growth, better recovery of tissue functionality and generally increasing the effectiveness of wound healing. The wound healing process is complicated, and factors such as burns or infections additionally interfere with the process and cause troublesome pain. Natural polysaccharide compounds, including chitosan (Ch) and hyaluronic acid (HA), are often used in medical and pharmaceutical applications as the starting materials for hydrogels [7,8,9]. Chitosan and hyaluronic acid are the basic ingredients for creating materials that mimic the extracellular matrix. Their use is beneficial due to biocompatibility, lack of toxicity, low tendency to cause allergies and relatively low price [5,6].

Chitosan has natural anti-inflammatory, antifungal and antibacterial effects. It also reduces the feeling of pain. Due to its good biocompatibility and biodegradability, chitosan-based dressing matrices are superior to other materials in promoting wound healing. Specifically, Ch can improve the proliferation and granulation of fibroblasts, stimulate neutrophils, affect membrane fluidity, enhance the analgesic and hemostatic effects, promote the activation of macrophages and the formation of extracellular matrix (ECM). In addition, chitosan has the ability to retain water, which is of great importance during the process of repair and regeneration of skin tissues [10]. At the same time, the ability to absorb excess inflammatory exudate allows the wound surroundings to be kept clean [11].

**Scheme 1:**
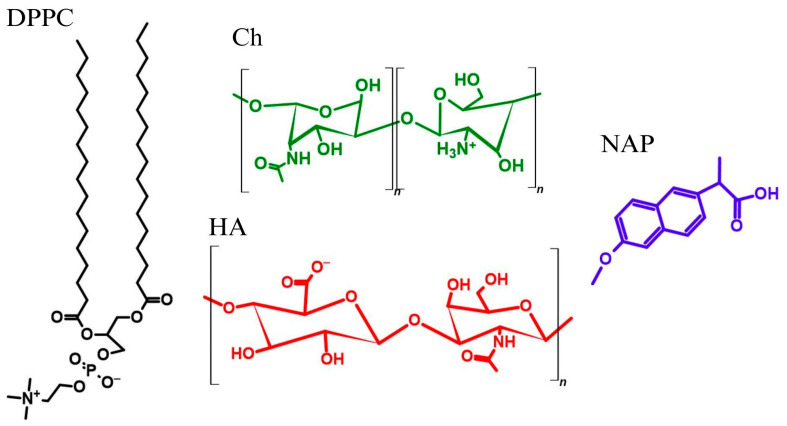
Structures of 1,2-dipalmitoyl-*sn*-glycero-3-phosphatidylcholine (DPPC), chitosan (Ch), hyaluronic acid (HA) and naproxen (NAP).

At acidic pH, the positively charged groups present in the structure of chitosan make it a cationic polyelectrolyte ($pK_{a}$ 6.1–6.5) [12]. The cationic nature of Ch promotes the interactions with a wide range of anionic glycosaminoglycans (GAGs). One of them is hyaluronic acid (HA) with a structure similar to chitosan (Scheme 1). HA as a natural skin component actively participates in many regulatory processes, such as cell proliferation, migration and differentiation [13]. As a highly hydrophilic biopolymer, it is able to absorb large amounts of water and form a loosely hydrated network that controls tissue hydration and water transport in the extracellular matrix [13]. HA is a polyanionic biopolymer ($pK_{a}$ 3.0) [14] that, when mixed with oppositely charged polycationic chitosan, creates polyelectrolyte complexes (PECs) through cooperative electrostatic interactions [15]. However, intermolecular forces are also engaged, including H-bonding, van der Waals forces, hydrophobic and dipole interactions [16]. In these complexes, the intrinsic properties of the individual compounds are preserved, but with the combination, additional benefits are obtained. Ch–HA hydrogels enable the cultivation of skin-matrix-producing cells (keratinocytes) and are effective in accelerating wound healing after skin ablation without causing side effects, such as inflammatory reactions and toxicity [17]. In principle, PEC hydrogels are well tolerated and can be employed in various applications. Their biocompatibility and sensitivity to modification, depending on environmental conditions, bring advantages as pharmaceutical excipients in drug delivery systems [8,16,18]. The beneficial effects of polysaccharides can also be synergistic with the delivered drugs [10].

In this article, the drug of interest is naproxen (NAP), which is one of the nonsteroidal anti-inflammatory drugs (NSAIDs) [19]. Naproxen is the drug of first choice for the treatment of mild to moderate pain and fever, soft tissue injuries and inflammatory disorders. It is widely used to control inflammation. Short-term usage of this drug accelerates wound healing by altering the levels of inflammatory cytokines. The anti-inflammatory effect of naproxen has been confirmed many times in recent decades and presented in detail in many papers [20,21,22]. NSAIDs are inhibitors of the pro-inflammatory enzyme cyclooxygenase (COX). This membrane-associated enzyme is involved in the conversion of arachidonic acid in prostaglandins, the hormones mediating the development of inflammatory reactions, which have a protective effect against injuries to the gastric mucosa [21]. The anti-inflammatory effect of NSAIDs results from lowering the threshold of pain receptor excitability. One of the major challenges associated with the use of NSAIDs is their poor gastrointestinal tolerance and a number of side effects related to abnormal stomach function [19,23]. The gastric effects of orally administered naproxen can be mitigated to some extent by its direct release from the polysaccharide hydrogel at the target site. Polysaccharides are effectively used as NAP carriers by forming a prodrug and offer a mechanism for improved drug efficacy and delivery. They have unique advantages, such as mucoadhesive properties and water storage capacity, which aid NAP loading across the cell membrane. Chitosan creates a gel barrier in an acidic environment, serving as an excipient and modulating substance in drug release systems [10,24]. The antibacterial nature of chitosan is notably advantageous. Hyaluronic acid, in turn, is particularly important in naproxen preparations applied directly to the skin. As a natural hydrophilic substance, it ensures adequate wound hydration and/or absorption of exudate [13,14]. A good solution could involve the inclusion of NAP in the composition of a hydrogel dressing and releasing the drug directly at the wound site. This provides the opportunity to significantly shorten the time it takes for the drug substance to penetrate biological membranes where the COX pathway begins and thus achieve better control over pain and wound healing.

NAP (Scheme 1) has the ability to change interactions in the hydrolipid layer of cell membranes. It interacts with either the apolar hydrocarbon chains and/or the polar headgroups of lipids affecting headgroup hydration. Therefore, it is important to conduct studies that will be a source of key information on the specific interactions of drugs with the lipid components of cell membranes in the presence of polysaccharides. Langmuir monolayers are widely used as a model for natural membranes to study such interactions between lipids, especially phospholipids, and various molecules of interest, such as drugs [25,26,27,28]. Phospholipids, as major constituents of the membrane, are responsible for its several properties like stability and semi-permeability. Eukaryotic cell membranes are mainly composed of phosphatidylcholines [29].

The aim of this paper is to characterize the physicochemical properties of 1,2-dipalmitoyl-*sn*-glycero-3-phosphatidylcholine (DPPC) (Scheme 1) monolayers with the addition of naproxen (NAP) on the polysaccharide subphase (Ch and/or HA). Since both polysaccharides have complementary functional groups (amino, carboxyl), the use of the Ch–HA mixture enables the formation of an ionic network, which is advantageous in the production of multi-network hydrogels with potential as an improved wound healing matrix. Polysaccharides themselves do not form Langmuir monolayers, but being components of the aqueous phase in direct contact with the membrane, they provide a mimicry of the cell/hydrogel interface behavior.

To achieve the aim, the Langmuir monolayer technique coupled with Brewster angle microscopy (BAM) was employed. Compression/adsorption isotherms and morphology images were registered at 20 °C. They allowed a determination of the effect of the subphase used on the elasticity (packing and ordering) of DPPC monolayers, their morphology and stability as a function of time. The anti-inflammatory activity of naproxen was not investigated directly. Our studies, however, focused mainly on the stage of delivering the drug to the site of pain in order to avoid side effects. Hence, we discussed in detail the effect of naproxen directly on model cell membranes. A potential mode of interactions between the phospholipid, drug and polysaccharides was identified to be responsible for changing the physicochemical properties of membranes. These interactions regulate the efficiency of drug delivery systems, including the controlled release of drug molecules, which can be crucial for a better understanding of the various biophysical mechanisms at the molecular level.

## 2. Results and Discussion

The properties of the DPPC monolayer in the absence and presence of NAP were tested on different subphases: ultrapure water (W), acetic acid solution (AA), chitosan solution (Ch), hyaluronic acid solution (HA), a mixture of chitosan and hyaluronic acid (Ch–HA). The effect of the selected compounds on the DPPC monolayer was assessed based on surface pressure versus mean molecular area (π−A) compression isotherms, surface pressure versus time (π−t) and area per molecule versus time (A−t) adsorption isotherms. The isotherm characteristics were supplemented with BAM morphology images.

### 2.1. (π − A) Compression Isotherms

**Figure 1:**
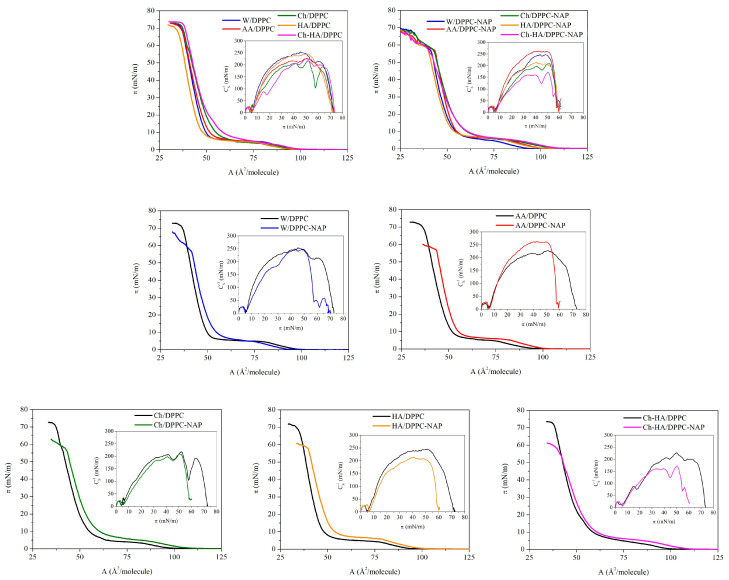
Surface pressure–area (π−A) compression isotherms and compression modulus–surface pressure ($C_{S}^{-1}-\pi$) dependencies obtained for DPPC monolayers in the absence and presence of naproxen (NAP) formed on the different subphases (W—water, AA—acetic acid, Ch—chitosan, HA—hyaluronic acid, Ch–HA—chitosan and hyaluronic acid mixture).

Figure 1 shows the surface pressure area per molecule (π−A) compression isotherms for the DPPC and DPPC–NAP monolayers formed on the W, AA, Ch, HA and Ch–HA subphases. Based on them, the compression modulus values were calculated and shown as a function of surface pressure in the form of insets. To better illustrate the effect of naproxen, the dependencies obtained for DPPC monolayers on individual subphases are presented separately.

The π−A isotherm for the W/DPPC (DPPC monolayer on water) displays a typical profile with a characteristic *plateau* reflecting a first-order phase transition from the liquid-expanded to the liquid-condensed (LE/LC) phase at π = 6.2 mN/m and inflection at the high surface pressure of 72 mN/m due to loss of the two-dimensional structure. The position of the compression isotherms changes noticeably with the addition of particular components to the water subphase. This is manifested by differences in the lift-off area ($A_{0}$), the limit area ($A_{lim}$), the LE/LC phase transition pressure ($\pi_{pt}$) and the collapse pressure ($\pi_{c}$). The values of these parameters are listed in Table 1.

Considering the DPPC monolayers without naproxen on different subphases, it can be seen that the presence of HA in the subphase reduces the lift-off area, thus shifting the isotherms toward smaller molecular areas (to the left side). On the contrary, chitosan expands the DPPC monolayers, causing an increase in the lift-off area and thus positioning the isotherms in the region of larger molecular areas (to the right side). Similar observations were noted previously [30,31,32]. This indicates the insertion of chitosan molecules between DPPC ones, with a simultaneous weakening of the attractive DPPC–DPPC forces. Interestingly, the expansion effect is enhanced for the Ch–HA mixture. This is due to the ability of these two polysaccharides to form complexes in water, with hyaluronic acid having the decisive influence due to its strong hydrophilic properties. In aqueous solution, HA forms a loosely hydrated network. Chitosan penetrates this structure, creating complexes through intermolecular forces, as well as hydrophobic and electrostatic interactions. This flexible structure allows for synergy of physicochemical properties compared to the characteristics of the individual components, as will be shown in the course of further considerations.

A similar trend of changes for Ch, HA and Ch–HA is observed for the $A_{lim}$ determined for closely packed DPPC monolayers on these subphases at high surface pressure values. This means that the polysaccharides are stably incorporated into the structure of the DPPC monolayer and are not pushed out of it even at a high compression state. Only for Ch/DPPC and HA–Ch/DPPC, the isotherms converge at surface pressures above 20 mN/m, indicating the dominant effect of Ch over HA on the DPPC monolayer properties. The collapse pressures ($\pi_{c}$) of all monolayers are similar and do not differ by more than 2.7 mN/m (Table 1). Importantly, the subphase components do not cause the disappearance of the LE/LC phase transition but only slightly affect the value of the surface pressure ($\pi_{pt}$) at which it occurs. The highest $\pi_{pt}$ value is obtained for Ch–HA/DPPC (9.5 mN/m), which points out that mixed polysaccharides hinder the formation of DPPC condensed phase domains the most in comparison to the individual components.

Naproxen itself does not form typical Langmuir monolayers at the liquid–gas interface. However, its presence in the interfacial region surrounded by film-forming DPPC molecules allows for the study of DPPC–NAP interactions using the Langmuir monolayer technique. The addition of naproxen influences the properties of the DPPC monolayer depending on the subphase composition (Figure 1, Table 1). Under the experimental conditions, naproxen occurs practically in an undissociated form ($pK_{a}$ 4.57) [33]; therefore, it has an affinity for the hydrocarbon tails of phospholipids [28]. In the initial stage of compression, NAP molecules are located between DPPC ones. This causes an increase in the area per DPPC molecule revealed by higher values of the $A_{0}$ parameter (Table 1). Only for the water subphase, the $A_{0}$ is lower. During further compression, NAP molecules migrate toward the hydrophobic region of the DPPC monolayers and remain there even at high surface pressure values. Therefore, a shift in the π−A isotherms toward larger molecular areas is observed for DPPC–NAP on all subphases (Figure 1). The area increase is also clearly visible in closely packed monolayers, as evidenced by the $A_{lim}$ values (Table 1). The smallest effect is observed on pure water ($A_{lim}$ increases by 1.7 ${Å}^{2}$), but the addition of polysaccharides intensifies it: Ch by 2.5 ${Å}^{2}$, HA by 5.1 ${Å}^{2}$ and Ch–HA by 3.4 ${Å}^{2}$ as compared to the monolayers without NAP. In this case, we are not dealing with a synergy effect for the Ch–HA mixture but only an average effect.

The collapse pressure ($\pi_{c}$) is significantly reduced by an average of 13 mN/m, indirectly pointing to lower stability of the DPPC–NAP monolayers. Meanwhile, the $\pi_{pt}$ increases by a value in the range of 0.5–2.9 mN/m, which clearly proves that naproxen hinders the verticalization of DPPC molecules to form condensed phase domains.

### 2.2. Compression Modulus

To characterize the physical state of the DPPC monolayers on different subphases and the possible phase transitions, the compression modulus ($C_{S}^{-1}$) was calculated by differentiating the π−A isotherms according to Equation (1):

(1) $$C_{S}^{-1}=-A{\left(\frac{\partial \pi }{\partial A}\right)}_{p,T}$$

where A denotes the mean molecular area; π is the surface pressure; p and T denote the conditions of constant atmospheric pressure and temperature, respectively.

The compression modulus is a parameter describing the elasticity of monolayers (degree of packing and ordering). The higher the $C_{S}^{-1}$ value, the lower the elasticity, and the greater the molecular packing and ordering.

The obtained values of compression modulus as a function of surface pressure are presented in Figure 1. Table 2 contains the maximal compression modulus values determined for the peaks before and after the phase transition and the compression modulus at the surface pressure of 30 mN/m, which corresponds to the internal lateral pressure of natural membranes. The maximal compression modulus characterizes the most tightly packed state of monolayers to which the limit area ($A_{lim}$) is assigned.

In agreement with previous studies [28,30], the elasticity of the DPPC monolayer on the water subphase is described by two $C_{S}^{-1}$ maxima separated by a minimum. This minimum corresponds to a *plateau* on the π−A isotherm and is associated with the LE/LC phase transition, whereas the maximal value of about 250 mN/m proves the condensed phase (LC) of the monolayer. The data for the compression modulus of the LC phase region ($C_{S}^{-1}$ above 100 mN/m) show a slight increase in the DPPC monolayer elasticity on all polysaccharide subphases, which is further enhanced by the presence of naproxen. The greatest reduction in the compression modulus ($C_{S,\;max2}^{-1}$) for the DPPC monolayer is observed in the presence of NAP on the mixed Ch–HA subphase (of 65 mN/m). Considering this parameter, we can again talk about the synergy effect when using two polysaccharides simultaneously. Meanwhile, individual polysaccharides reduce $C_{S}^{-1}$ to a lesser extent—by 11 mN/m and 31 mN/m for Ch and HA, respectively. Taking into account the elasticity at 30 mN/m (Table 2), the changes caused by the particular polysaccharides are very similar to those obtained at $C_{S,\;max2}^{-1}$, while the decrease in $C_{S}^{-1}$ for the Ch–HA mixture is smaller, amounting to 17 mN/m. This means that the elasticity of Ch–HA/DPPC–NAP increases drastically at surface pressures above the corresponding internal pressure of the natural membranes. Then, there is a greater disruption of the DPPC molecular packing by both polysaccharides and NAP. The decrease in $C_{S}^{-1}$ caused by NAP is similar to that previously reported by Przykaza et al. [28]. The loose polysaccharide network allows for the diffusion of water and/or drug molecules. This is important for wound care applications, where the high water content keeps the skin well hydrated, and the flexibility of the network allows for precise modulation of drug release [8]. The obtained results once again confirm the leading effect of Ch–HA flexible structure in this type of system with potential applications as a drug delivery.

### 2.3. Morphology

**Figure 2:**
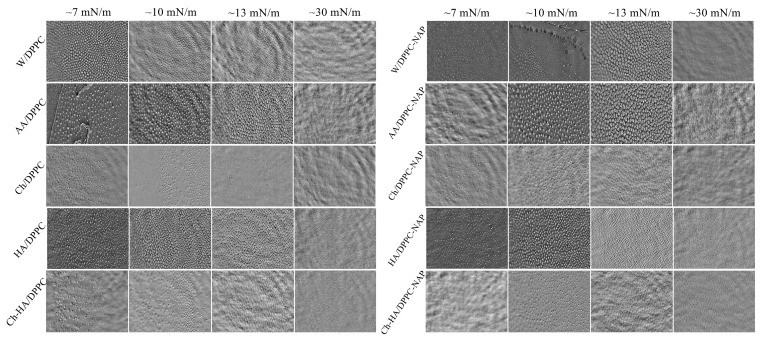
BAM images taken at different stages of DPPC monolayer compression in the absence and presence of naproxen (NAP) on the different subphases (W—water, AA—acetic acid, Ch—chitosan, HA—hyaluronic acid, Ch–HA—chitosan and hyaluronic acid mixture).

For a more in-depth π−A isotherm analysis, real-time observation of surface morphology for the DPPC monolayer on different subphases was performed using Brewster angle microscopy (BAM). Imaging of the phase behavior at different stages of monolayer compression was performed. Figure 2 presents the recorded BAM images showing the surface fragments with dimensions of 720 × 400 μm^2^.

The DPPC monolayer exhibits a morphology known as “cloverleaf shape” in the LE/LC phase transition region. The LC domains begin to rise and adopt a well-defined shape at a low surface pressure of about 7 mN/m (Figure 2). The lobed shape of the closely packed domains is due to the chiral nature of the DPPC molecules, providing direct visual evidence of a long range orientational order [34]. With compression above the LE/LC phase transition, the domains merge, and their structure successively blurs, reaching homogeneity at ca. 30 mN/m. When the polysaccharides are included in the subphase, the monolayer morphology only subtly changes, as revealed by the similar shape and size of the domains. However, NAP induces a larger disturbance in morphology by delaying the growth of domains and their integration into a uniform monolayer. At a given pressure, the domains are smaller and less developed (Figure 2). This observation correlates with the increased values of the LE/LC phase transition pressure ($\pi_{pt}$, Table 1). As shown above, the subphase components affect the molecular organization in the condensed DPPC monolayer, making it less packed. These changes suggest that Ch, HA and/or NAP are incorporated into the DPPC monolayer structure, weakening the attractive interactions between phospholipid molecules. This, in turn, hinders the arrangement of DPPC molecules in a vertical position conducive to the emergence of condensed domains during the LE/LC phase transition, and ultimately, the formation of a homogeneous film.

Accordingly, it can be said that neither the polysaccharides nor naproxen disturb the structure of the DPPC monolayer significantly, which can directly translate into maintaining the proper functioning of the phospholipid membrane, indicating their biocompatibility. The membranes preserve adequate mechanical integrity while providing a fluid environment in which diffusion and interaction processes can occur. Therefore, the combination of these compounds could be used in preparations intended for close proximity to cell membranes, e.g., on the skin.

### 2.4. Stability over Time of DPPC Monolayers Formed on Different Subphases Without External Force (Relaxation)

**Figure 3:**
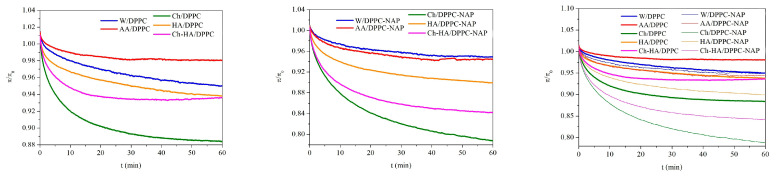
Dependence of normalized surface pressure changes ($\pi /\pi_{0}$) on time for the DPPC monolayers in the absence and presence of naproxen (NAP) on the different subphases (W—water, AA—acetic acid, Ch—chitosan, HA—hyaluronic acid, Ch–HA—chitosan and hyaluronic acid mixture).

In order to determine the stability of the DPPC monolayers over time, surface pressure changes were recorded during 60 min of relaxation on different subphases. Relaxation measurements were started after prior compression of the monolayer to a surface pressure of 30 mN/m and stopping the barriers that did not compress for longer. Figure 3 shows the normalized surface pressure changes ($\pi /\pi_{0}$) over time to eliminate the effect of any material loss before the target surface pressure is reached.

Depending on the subphase composition, the DPPC monolayers reveal distinct responses to mechanical compression. For all monolayers, a decrease in surface pressure over time is observed, which is an indicator of reduced stability due to the ejection of molecules from the interface. A clear decrease is visible within the first 10–15 min of relaxation, from 1% for AA/DPPC to 9% for Ch/DPPC (Figure 3). As the experiment progresses, the rate of decline slows down, and finally, the $\pi /\pi_{0}$ changes are very slight, running parallel to the time axis, pointing out monolayer stability. After one hour of relaxation, the highest $\pi /\pi_{0}$ reduction of 12% was obtained for Ch/DPPC, indicating the lowest stability of the DPPC monolayer on the chitosan subphase. The results confirm that chitosan molecules exhibit high affinity for DPPC ones and migrate into and/or accumulate under the monolayer, leading to its local destabilization and desorption of some phospholipid molecules from the liquid–air interface to the subphase. Therefore, the area per molecule in the DPPC monolayer diminishes, although the barriers’ position is kept fixed. This correlates with the decreased surface pressure. Meanwhile, another polysaccharide (HA) causes a $\pi /\pi_{0}$ drop that is half as large, reaching 6% after an hour. Importantly, the $\pi /\pi_{0}$ changes for the Ch–HA mixture are intermediate between those for Ch and HA (Figure 3), which means that the combination of Ch–HA provides better DPPC monolayer stability than Ch alone and is comparable to HA/DPPC after 1 h. In this case, the use of both polysaccharides simultaneously in defined proportions is the most beneficial and can be optimal regarding specific applications. For instance, a kind of synergy between hyaluronic acid (HA) and phospholipid DPPC was reported for a lubricating layer [35,36]. It was shown that HA/DPPC composite layers have better stability than pure DPPC layers at high hydrostatic pressures. These spontaneously self-assembled structures can give rise to a self-healing ability.

Similar trends are observed in the presence of naproxen, but NAP magnifies the effect of polysaccharides on the stability of the DPPC monolayer. After one hour of relaxation at an initial surface pressure of 30 mN/m, a $\pi /\pi_{0}$ drop of 20% is obtained for Ch/DPPC–NAP, 7% for HA/DPPC and 15% for Ch–HA (Figure 3). HA prevents desorption of monolayer molecules to the bulk phase to the greatest extent, while Ch enhances this process. Again, the use of the Ch–HA mixture balances this effect, indicating competitive interactions of polysaccharides with DPPC. Depending on the subphase, the more destabilizing effect of NAP is manifested in a further $\pi /\pi_{0}$ decrease of 8% (Ch/DPPC–NAP), 1% (HA/DPPC–NAP) and 9% (Ch–HA/DPPC–NAP) compared to the corresponding systems without NAP (Figure 3). Nevertheless, one-hour relaxation leads to practically constant surface pressure values, with negligible loss of material from the interface, thus demonstrating satisfactory stability of the monolayers on the studied subphases.

### 2.5. Stability over Time of DPPC Monolayers Formed on Different Subphases Under the Influence of an External Force (Oscillation)

**Figure 4:**
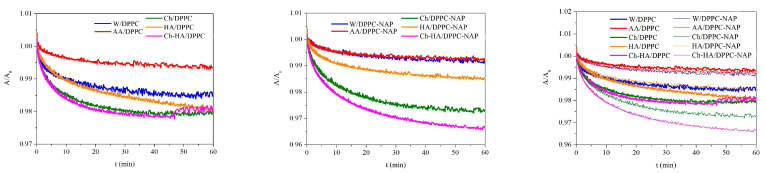
Dependence of normalized area per molecule ($A/A_{0}$) on time for the DPPC monolayers in the absence and presence of naproxen (NAP) at a constant surface pressure of 30 mN/m on the different subphases (W—water, AA—acetic acid, Ch—chitosan, HA—hyaluronic acid, Ch–HA—chitosan and hyaluronic acid mixture).

Another way of determining the stability of DPPC monolayers on different subphases was based on changes in the mean molecular area at a constant surface pressure (30 mN/m) maintained by oscillating movements of the barriers at a constant rate during a specific period of time (1 h). The obtained relative values of area per molecule ($A/A_{0}$) as a function of time are presented in Figure 4.

The molecular packing density in the monolayer at the selected surface pressure of 30 mN/m is consistent with that of bilayers and biomembranes in which there is an equivalent internal lateral pressure [37]. The increase in the area per molecule can be ascribed to incorporation of the subphase components into the monolayer structure, making it more expanded (less packed). On the contrary, the decrease in the area per molecule clearly demonstrates the loss of molecules from the interface into the bulk.

The results evidently show only a 2% reduction in the mean molecular area within one hour of equilibration on the polysaccharide subphases (Ch/DPPC, HA/DPPC and Ch–HA/DPPC), while for the W/DPPC, the reduction is 1.5%, and for AA/DPPC, it is only 0.5% (Figure 4). The above data indicate a high stability of the studied systems subjected to a constant external force. The packing density of the DPPC molecules changes slightly. It can be expected that such small changes in molecular packing will not have a significant impact on the biological functions of membranes.

In the presence of NAP, the A−t dependences are rather more differentiated, and a 3% reduction in the molecular area is obtained for Ch–HA/DPPC–NAP, 2% for Ch/DPPC–NAP and 1.5% for HA/DPPC–NAP. For the water and AA subphases, the $A/A_{0}$ changes are the smallest (0.5%) and similar to each other. Therefore, it can be stated that the decisive factor responsible for the little loss of DPPC molecules is their interactions with polysaccharides, which pull phospholipid molecules into the subphase. For the DPPC monolayers with naproxen, the difference in the mean molecular area is around 1% compared to those without naproxen. These studies distinctly confirm the stability of the tested systems. Therefore, the use of chitosan in combination with hyaluronic acid widely present in the skin matrix is a rational approach to improving wound healing. Their intrinsic properties can protect the wound, accelerate tissue regeneration and prevent bacterial infections.

### 2.6. Possible Model of Interactions at the Air–Liquid Interface

It is known that naproxen, as a nonsteroidal anti-inflammatory drug (NSAID), is a nonspecific inhibitor of the cyclooxygenase (COX) pathway, thereby inhibiting the formation of inflammatory mediators, such as prostaglandins. The transformation of arachidonic acid, which initiates the inflammatory pathway, is a membrane-related process [21] and should depend on both the physicochemical properties of the membrane and the changes resulting from drug binding. Since COX is a membrane-associated enzyme, the study of membrane parameters before and after naproxen administration indirectly provides information on the anti-inflammatory effect of the drug. These membrane–drug interactions can be further modified in the presence of polysaccharides, often used in drug delivery systems and hydrogel-based dressings. Therefore, understanding the physicochemical properties of lipid membranes is crucial in the context of both their biological functions and potential applications.

**Scheme 2:**
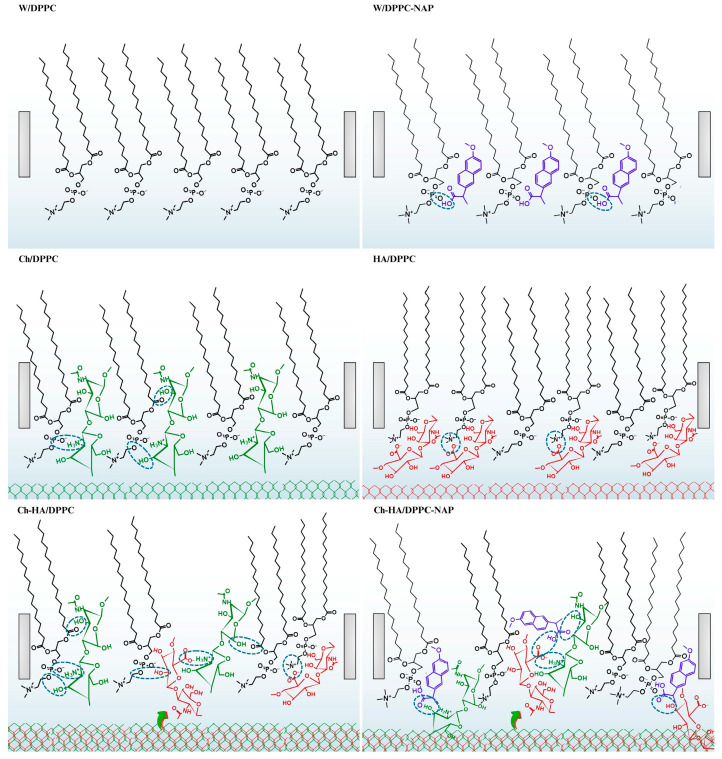
Possible models of interactions between DPPC, naproxen (NAP) and polysaccharides (Ch—chitosan, HA—hyaluronic acid, Ch–HA—chitosan and hyaluronic acid mixture).

DPPC molecules on the aqueous subphase form a condensed LC monolayer in which all-trans chains are tilted with respect to the normal to the plane of the subphase (Scheme 2). The tilt is caused by compensating for the mismatch in spatial requirements between the head and the tail. The heads are relatively large and impose an area occupied by the molecule in the LC phase, which is about 41 ${Å}^{2}$ in the most closely packed state (Table 1). This value is larger than the minimum cross-sectional area of two PC hydrocarbon chains, which is 38 ${Å}^{2}$ [38]. As revealed by Ma and Allen [38], the three methyl groups of choline are tilted from the surface normal and aligned slightly parallel to the air–water interface. Furthermore, the phosphate group is much less hydrated in the LC phase because water is squeezed out of the hydration shell of the headgroup during compression [38]. This can promote the formation of hydrogen bonds with other components of the subphase when they are present at the interface. This process can be aided by the fact that zwitterionic DPPC molecules interact via Lifshitz–van der Waals forces and do not form hydrogen bonds with each other.

Let us now consider the interactions between HA and DPPC molecules (Scheme 2). HA possesses affinity for the DPPC monolayer and is adsorbed at its surface via hydrophobic interactions and the formation of hydrogen bonds. Molecular dynamic simulations by Li et al. [36] showed that H-bonds are preferentially formed by the hydroxyl, ether and amide groups of HA. However, due to the lowest steric hindrance, the hydroxyl oxygen at the methylene group ($-CH_{2}-OH$) is most often the hydrogen atom donor. The acceptor is mainly the oxygen atoms of the phosphate group of DPPC but also the oxygen atoms of the carbonyl groups (>C=O) located at the acyl chains. In our studies, when the monolayer is expanded (at large areas per molecule), the hydrophobic interactions of the >$CH_{2}$ and $-CH_{3}$ groups between HA and DPPC can be more important. However, during compression, when DPPC molecules become vertical, directing their chains toward the air, HA can be partially pushed out of the monolayer, accumulating in the headgroup region. In such case, both hydrogen bonding and electrostatic forces contribute. Negatively charged HA groups interact readily with the positively charged choline groups of DPPC exposed toward the subphase, thereby changing the orientation of the phosphocholine groups to a more vertical one. This causes a decrease in the area per molecule in the DPPC monolayer and a shift in the π−A compression isotherm to the left side with respect to that of DPPC/W and DPPC/AA (Figure 1, Table 1). Secondly, HA can form larger aggregates than Ch, and therefore, access to the phospholipid hydrophobic chains in the condensed monolayers is more difficult.

In line with the studies by Pavinatto et al. [31,32], chitosan adsorbs at the interface, causing the expansion of the DPPC monolayer by interacting via electrostatic and hydrophobic forces. The latter lead to incorporation of chitosan into the hydrophobic tails of the phospholipid. With compression, chitosan is progressively pushed out of the monolayer, but even at high surface pressures (in small areas), it remains at the interface, forming a subsurface layer. This is mainly ensured by electrostatic interactions with the DPPC headgroups (Scheme 2), which makes the monolayer more flexible, as evidenced by the reduced compression modulus (Figure 1, Table 2).

As demonstrated above, the changes in the structure and packing of the DPPC monolayer that occurred during its contact with the mixed Ch–HA subphase are greater than those caused by individual components. Taking into account all the physicochemical properties tested, this synergistic effect is manifested by the higher $A_{0}$, $A_{lim}$, $\pi_{c}$ and $\pi_{pt}$ values and lower $C_{S}^{-1}$ values (Table 1 and Table 2). Under the experimental conditions (pH 3.3–3.5), chitosan is positively charged ($pK_{a}$ 6.1–6.5) [12], hyaluronic acid—negatively charged ($pK_{a}$ 3.0) [14], DPPC—zwitterionic and NAP—neutral ($pK_{a}$ 4.57) [31]. As mentioned previously, Ch and HA are two oppositely charged macromolecules; they can self-assemble into polyelectrolyte Ch–HA complexes upon mixing due to polyanion–polycation electrostatic interactions held between the ammonium groups of chitosan and the carboxylate groups of HA [15]. Furthermore, both polysaccharides are capable of interacting through interchain H-bonds and hydrophobic interactions involving hydroxyl groups and the hydrocarbon skeleton, respectively (Scheme 2) [7,39]. These non-electrostatic intermolecular forces become more important after mutual neutralization of polysaccharides (reduction in electrostatic repulsion) and can even be dominant in systems with zwitterionic DPPC and/or non-ionized NAP.

Moreover, in the presence of NAP, the shift in the π−A isotherms for the DPPC monolayers to the region of larger areas per molecule is observed on all subphases (Figure 1). The lower $C_{S}^{-1}$ values evidence looser packing of the DPPC–NAP monolayer, which is indicative of the penetration of NAP molecules between DPPC ones (Scheme 2). The changes are enhanced by polysaccharides. This synergy is mostly related to specific interactions; therefore, subphase components penetrate the monolayer and remain there, even when it is closely packed (Figure 1, Table 2).

According to the FTIR–ATR results obtained by Manrique-Moreno et al. [40], naproxen is located preferentially in the polar headgroups of the phospholipids, close to the phosphate region. Interactions through carbonyl and amino groups for phosphatidylcholine–naproxen (PC–NAP) are hardly possible. This sheds light on our findings. We can assume that the carboxyl group of the drug is stationed near the phosphate region of DPPC, interacting via O–H–O hydrogen bonding, which is competitive for the formation of hydrogen bonds with the water molecules of the hydration shell (Scheme 2). The van der Waals attraction between the hydrocarbon chains of DPPC is weakened, and therefore, the acyl chains are more exposed to the polar–apolar interface. In such situation, the aromatic rings of neutral NAP molecules are most likely inserted into the acyl chain region of the DPPC monolayer, interacting via hydrophobic forces.

In this regard, it is conceivable that the large aromatic groups of NAP could act as a steric hindrance. The drug causes an evident fluidization of the membrane due to changes in the packing density in the acyl chain region, headgroup region and the surface-bound water molecules. By improving the fluidity of membrane models, NAP can increase their permeability, as reported by Pereira-Leite et al. [41]. NAP being a drug for pain, inflammation and fever, to achieve its target, the enzyme cyclooxygenase, it must diffuse across cell membranes, which can interfere with the membrane properties. Polysaccharides are capable of interacting with cell membranes to weaken the tightly coupled barrier action and to assist the loading of drugs across the cell membrane [10]. Hence, polysaccharides promoting membrane permeability can allow for better drug penetration to enhance the therapeutic effect. In other words, a stable, yet flexible, membrane, which, owing to the addition of polysaccharides, allows the drug to flow freely, gives hope for effective anti-inflammatory action while avoiding side effects (e.g., injuries to the gastric mucosa).

Alterations to the hydration shell fulfill a significant function in the stability of the membrane. The adsorption isotherms performed at the surface pressure of 30 mN/m indicated high stability of the DPPC monolayers both in the relaxation process and when subjected to a constant external force (Figure 3 and Figure 4). It is likely that the hydrogen bonds formed among DPPC–NAP–polysaccharide molecules contribute mainly to the stability of the membrane structure by compensating for lost hydrogen bonds with water when the naproxen and/or polysaccharide molecules are in close proximity to the DPPC monolayer surface. Moreover, hydrophobic forces seem to be essential for such type of association. Accordingly, the strong interactions between polysaccharides, NAP and DPPC suggest that NAP may be a good candidate for targeted drug delivery to pain sites from a polysaccharide matrix while avoiding direct local interactions with the gastric lining. In addition, the physicochemical properties of polysaccharides complement each other in this aspect: they are antibacterial, hydrophilic and have the ability to maintain proper wound hydration while absorbing exudate. Using these components individually, such benefits would not be possible.

## 3. Materials and Methods

### 3.1. Materials

1,2-Dipalmitoyl-*sn*-glycero-3-phosphatidylcholine (DPPC) was purchased from Sigma-Aldrich (Saint Louis, MO, USA), naproxen (NAP) from Cayman Chemical Company (Ann Arbor, MI, USA), chitosan (MW 100,000–300,000 Da; deacetylation degree 82%) from Acrōs Organics (Geel, Belgium). Hyaluronic acid (HA) was a commercial 1% solution. Acetic acid (99.5–99.9%) was purchased from Avantor Performance Materials Poland S.A. (Gliwice, Poland), chloroform from Chempur (Piekary Śląskie, Polska) and methanol from Romil Pure Chemistry (Cambridge, UK). The purity of all reagents was ≥99.0%. A chloroform and methanol mixture (4:1, *v*/*v*) was employed as a solvent for DPPC and NAP. The solutions were prepared at a concentration of 1 mg/mL. DPPC and NAP were mixed at a molar ratio of 1:1. Ultrapure Milli-Q water (resistivity 18.2 MΩcm, surface tension 72.8 mN/m at 20 °C and pH 5.6) was used as a subphase (W) and for aqueous solution preparation: 0.1% acetic acid solution (AA), 0.1 mg/mL chitosan solution in 0.1% acetic acid (Ch), 0.5 mL/L hyaluronic acid solution in 0.1% acetic acid (HA). The mixed subphase (Ch–HA) was produced by simply mixing these two polysaccharide solutions, keeping polysaccharide concentrations the same as in the basic solutions. The pH of the acidic subphases was 3.3–3.5.

### 3.2. Methods

#### 3.2.1. Compression Isotherms

A computer-controlled Langmuir–Blodgett trough (KSV NIMA, Biolin Scientific, Stockholm, Sweden) equipped with two movable barriers was used to form the Langmuir monolayer of DPPC and DPPC–NAP. The trough was maintained at a temperature of 20 °C ± 0.1 °C using a water circulating thermostat (Lauda, Schwechat, Austria). Additionally, an anti-vibration system was applied to counteract vibrations, and a cover protected against the effects of air movement and dust contamination. Surface pressure was measured using a platinum Wilhelmy plate method, with an accuracy of ±0.1 mN/m. Surface pressure (π) was the difference between the surface tension of the bare subphase and the subphase with the monolayer. After thorough cleaning, the trough was filled with the given subphase (W, AA, Ch, HA, Ch–HA). Then, 60–80 µL of DPPC or DPPC/NAP solution was applied to the subphase surface using a Hamilton microsyringe (Hamilton, Bonaduz, Switzerland), precision ± 1.0 μL. The solvents were allowed to evaporate for 10 min, and the monolayer was symmetrically compressed at a rate of 10 mm/min until it collapsed. The surface pressure–mean molecular area (π –A) compression isotherms were registered at constant temperature in three independent series. The uncertainty of the molecular areas was no more than 2 ${Å}^{2}$/molecule.

#### 3.2.2. Monolayer Morphology

Simultaneously, the monolayer morphology was monitored using the Brewster angle microscope (BAM, nanofilm_ultrabam) from Accurion GmbH (Göttingen, Germany). The system was coupled to the Langmuir–Blodgett trough and enabled real-time visualization during monolayer compression. The BAM had a built-in 50 mW laser emitting *p*-polarized light with a wavelength of 658 nm that was reflected from the liquid/air interface at a Brewster angle of 53.2°. BAM images were processed for better contrast by scaling the brightness of each image and removing the background. The size of BAM images was 720 × 400 μm^2^.

#### 3.2.3. Adsorption Isotherms

The stability of DPPC and DPPC–NAP monolayers on different subphases (W, AA, Ch, HA, Ch–HA) was checked in two ways: (1) surface pressure versus time (π−t) and (2) area per molecule versus time (A−t) changes. In both cases, the same procedure of monolayer preparation was used as for the compression isotherms. In the first case (1), the spreading monolayers were compressed to a surface pressure of 30 mN/m and then equilibrated for one hour at a constant barrier position (no oscillations). Stability was assessed based on changes in the surface pressure as a function of time (π−t) without an external force acting on the monolayer (relaxation). In the second case (2), the compressed monolayer was kept at a constant surface pressure of 30 mN/m for one hour by oscillating the barriers at a constant speed (5 mm/min). The changes in area per molecule versus time (A−t) were registered.

## 4. Conclusions

NAP and/or polysaccharides (Ch, HA, Ch–HA) were found to affect the Langmuir monolayers of DPPC, employed here as a simplified membrane model. The effect was revealed by shifting the π−A isotherms and increasing the monolayer elasticity. Surface morphology (BAM images) showed that the insertion of additional components to the subphase and/or the interface retarded the formation of the condensed phase by hindering the verticalization of DPPC molecules. Interestingly, the mixture of polysaccharides (Ch–HA) had a stronger effect than each of the polysaccharides separately. The fluidizing effect of polysaccharides, particularly noticeable for Ch and HA used simultaneously, was enhanced in the presence of naproxen. Such a synergistic effect can be ascribed to more extensive interactions in the DPPC headgroup region, also involving the formation of an increased number of hydrogen bonds with water molecules, which makes the monolayer more expanded. Alterations to the hydration shell fulfill a significant function in the stability of the membrane. In addition, oppositely charged Ch and HA can associate in polyelectrolyte complexes, possibly occupying larger areas than Ch and HA individually and thus contributing to larger DPPC monolayer expansion. These types of flexible complexes increase the permeability of cell membranes, which can lead to better drug penetration and enhanced therapeutic effect.

In our opinion, the studies conducted provide essential information on the behavior of membrane models with embedded naproxen in the polysaccharide environment at the molecular level and significantly facilitate the understanding of the entire complex process of interactions in the membrane–drug–polysaccharide system in many specific applications.

## Figures and Tables

**Table 1:** The lift-off area ($A_{0}$), the limit area ($A_{lim}$), the LE/LC phase transition pressure ($\pi_{pt}$) and the collapse pressure ($\pi_{c}$) values obtained for DPPC monolayers in the absence and presence of naproxen (NAP) formed on the different subphases (W—water, AA—acetic acid, Ch—chitosan, HA—hyaluronic acid, Ch–HA—chitosan and hyaluronic acid mixture). All data are reported as the mean ± standard deviation (SD).

DPPC	W	AA	Ch	HA	Ch–HA
$A_{0}$ $\;[{Å}^{2}]$	96.4 ± 1.3	93.3 ± 1.2	97.5 ± 1.3	92.7 ± 1.2	98.8 ± 1.3
$A_{lim}$ $\;[{Å}^{2}]$	41.4 ± 0.9	41.0 ± 0.9	42.1 ± 0.9	38.4 ± 0.8	42.7 ± 1.0
$\pi_{pt}$ [mN/m]	6.2 ± 0.1	6.9 ± 0.1	5.3 ± 0.1	6.6 ± 0.1	9.5 ± 0.1
$\pi_{c}$ [mN/m]	72.6 ± 0.6	72.6 ± 0.6	72.6 ± 0.6	70.5 ± 0.6	73.1 ± 0.6
DPPC–NAP					
$A_{0}$ $\;[{Å}^{2}]$	91.1 ± 1.2	99.2 ± 1.3	107.5 ± 1.4	99.6 ± 1.3	108.6 ± 1.4
$A_{lim}$ $\;[{Å}^{2}]$	43.1 ± 1.0	46.0 ± 1.0	44.6 ± 1.0	43.5 ± 0.9	46.1 ± 1.0
$\pi_{pt}$ [mN/m]	7.5 ± 0.1	7.4 ± 0.1	8.2 ± 0.1	7.9 ± 0.1	8.4 ± 0.1
$\pi_{c}$ [mN/m]	63.4 ± 0.5	58.0 ± 0.5	57.6 ± 0.5	58.2 ± 0.5	60.2 ± 0.5

**Table 2:** The maximal compression modulus values determined for the peaks before ($C_{S,\;max1}^{-1}$) and after ($C_{S,\;max2}^{-1}$) the phase transition and the compression modulus at the surface pressure of 30 mN/m ($C_{S,\;30}^{-1}$) obtained for DPPC monolayers in the absence and presence of naproxen (NAP) formed on the different subphases (W—water, AA—acetic acid, Ch—chitosan, HA—hyaluronic acid, Ch–HA—chitosan and hyaluronic acid mixture). All data are reported as the mean ± standard deviation (SD).

DPPC	W	AA	Ch	HA	Ch–HA
$C_{S,\;max1}^{-1}$ [mN/m]	26.0 ± 0.4	22.4 ± 0.3	19.9 ± 0.3	23.5 ± 0.4	25.1 ± 0.4
$C_{S,\;max2}^{-1}$ [mN/m]	253.5 ± 4.0	227.3 ± 3.6	207.8 ± 3.3	245.2 ± 3.9	226.1 ± 3.6
$C_{S,\;30}^{-1}$ [mN/m]	226.1 ± 4.4	201.1 ± 3.9	189.2 ± 3.7	219.8 ± 4.3	170.9 ± 3.3
DPPC–NAP					
$C_{S,\;max1}^{-1}$ [mN/m]	24.7 ± 0.4	27.9 ± 0.4	21.9 ± 0.3	27.9 ± 0.4	24.3 ± 0.4
$C_{S,\;max2}^{-1}$ [mN/m]	247.2 ± 3.9	261.8 ± 4.1	197.1 ± 3.1	213.8 ± 3.4	161.4 ± 2.6
$C_{S,\;30}^{-1}$ [mN/m]	185.2 ± 3.6	231.1 ± 4.5	177.7 ± 2.8	188.8 ± 3.7	153.5 ± 3.0

## Data Availability

Data are contained within the article.
